# Supplementary material for: The NANOG Transcription Factor Induces Type 2 Deiodinase Expression and Regulates the Intracellular Activation of Thyroid Hormone in Keratinocyte Carcinomas
Source: Cancers (Basel). 2020 Mar 18;12(3):715. doi: 10.3390/cancers12030715 (PMC7140064; doi:10.3390/cancers12030715)
Supplement: Supplementary file 1 [file cancers-12-00715-s001.pdf]

Supplementary Materials: The NANOG Transcription Factor Induces Type 2 Deiodinase Expression and Regulates the Intracellular Activation of Thyroid Hormone in Keratinocyte Carcinomas

Annarita Nappi, Emery Di Cicco, Caterina Miro, Annunziata Gaetana Cicatiello, Serena Sagliocchi, Giuseppina Mancino, Raffaele Ambrosio, Cristina Luongo, Daniela Di Girolamo, Maria Angela De Stefano, Tommaso Porcelli, Mariano Stornaiuolo and Monica Dentice

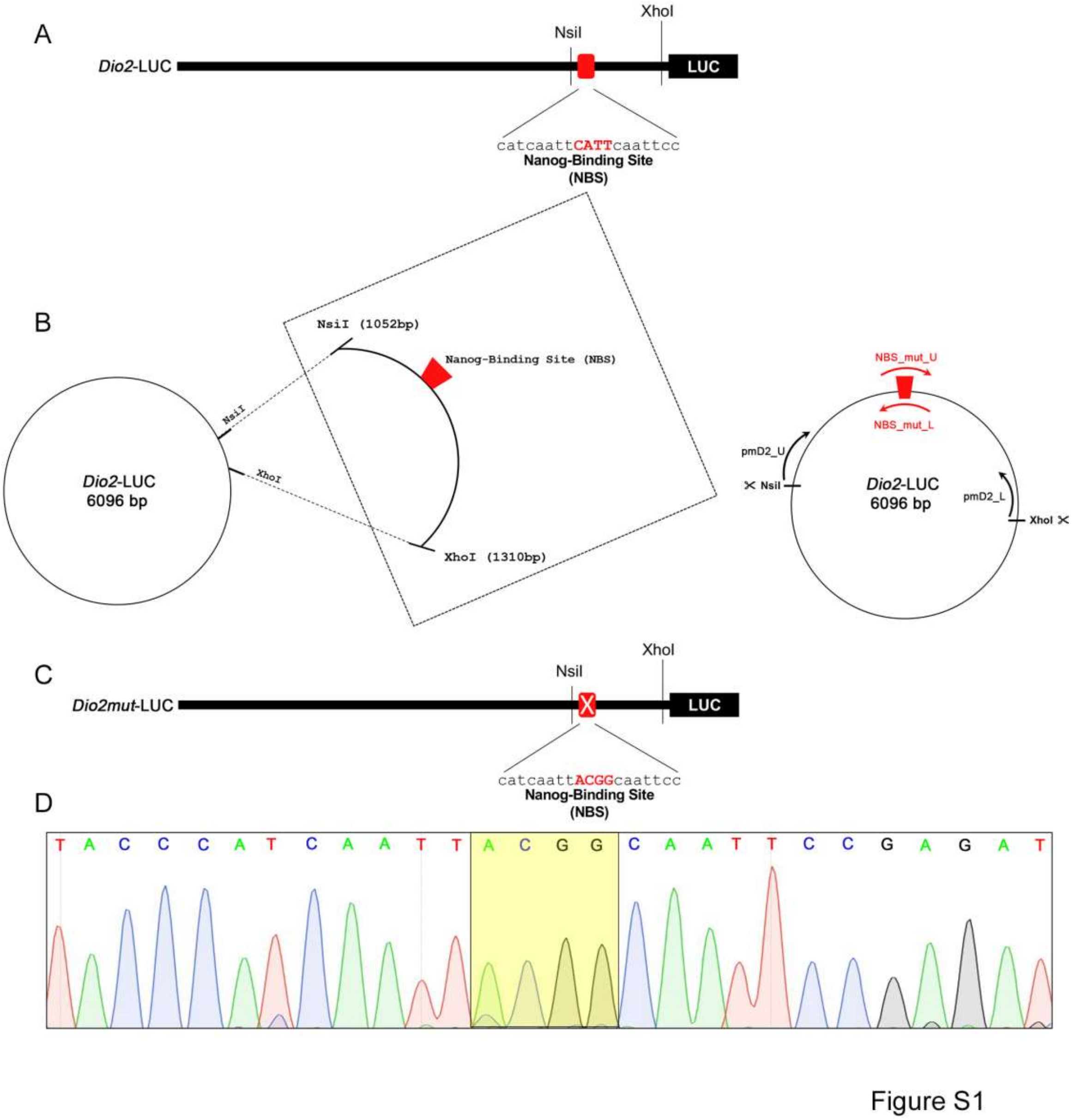

Figure S1. Strategy for the mutagenesis of Dio2 promoter. (**A**) Schematic representation of NANOG Binding Site within the *Dio2* promoter region. (**B**) Schematic diagram for site-directed mutagenesis of NANOG Binding Site on *Dio2* promoter region by Recombinant PCR. (**C**) Representation of the mutated NANOG Binding Site on *Dio2* promoter region. (**D**) Electropherogram of the NANOG Binding Site mutation within the *Dio2* promoter.

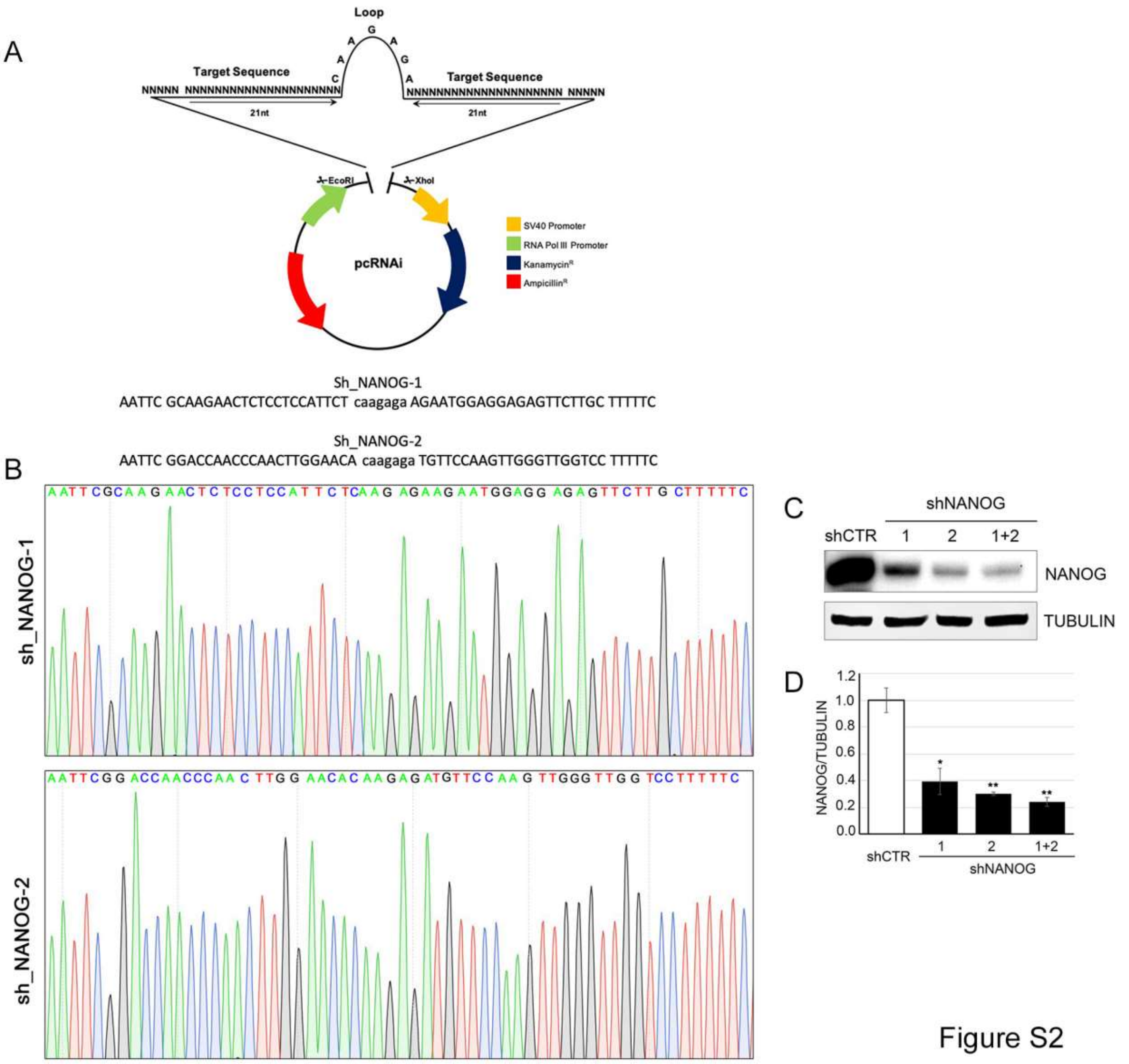

Figure S2. Strategy for the silencing of NANOG expression. (**A**) Cloning strategies for the generation of NANOG shRNA expression vectors. (**B**) Electropherograms of the NANOG shRNA sequences cloned into pcDNA3.1 vector. (**C**) Validation of effective NANOG down-modulation by two different NANOG shRNA vectors was assessed by Western Blot analysis of NANOG expression in BCC cells. (**D**) Quantification of NANOG protein levels versus Tubulin levels in the same experiment as in C is represented by histograms.

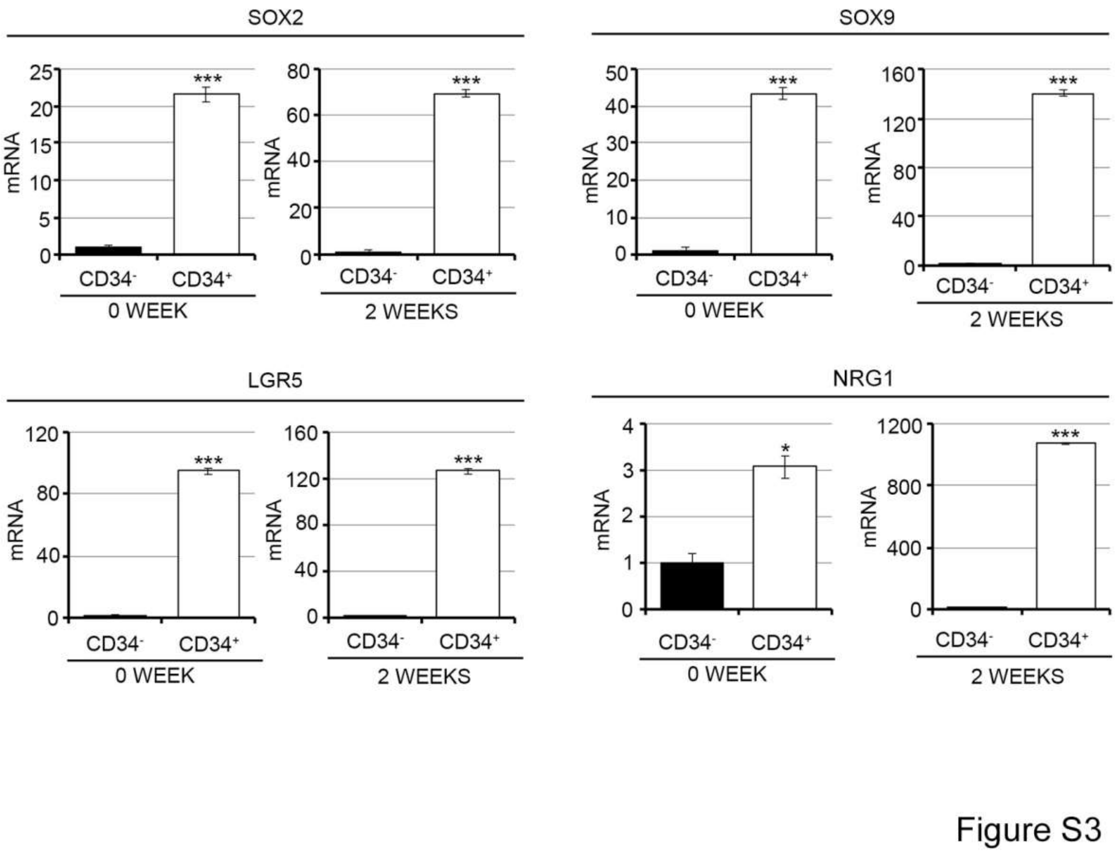

Figure S3. The CD34+ cells are characterized by the expression of typical epithelial stemness genes. The mRNA levels of a panel of indicated stemness markers of epidermis were measured by Real Time PCR in the same experiment indicated in figure 3F and G.

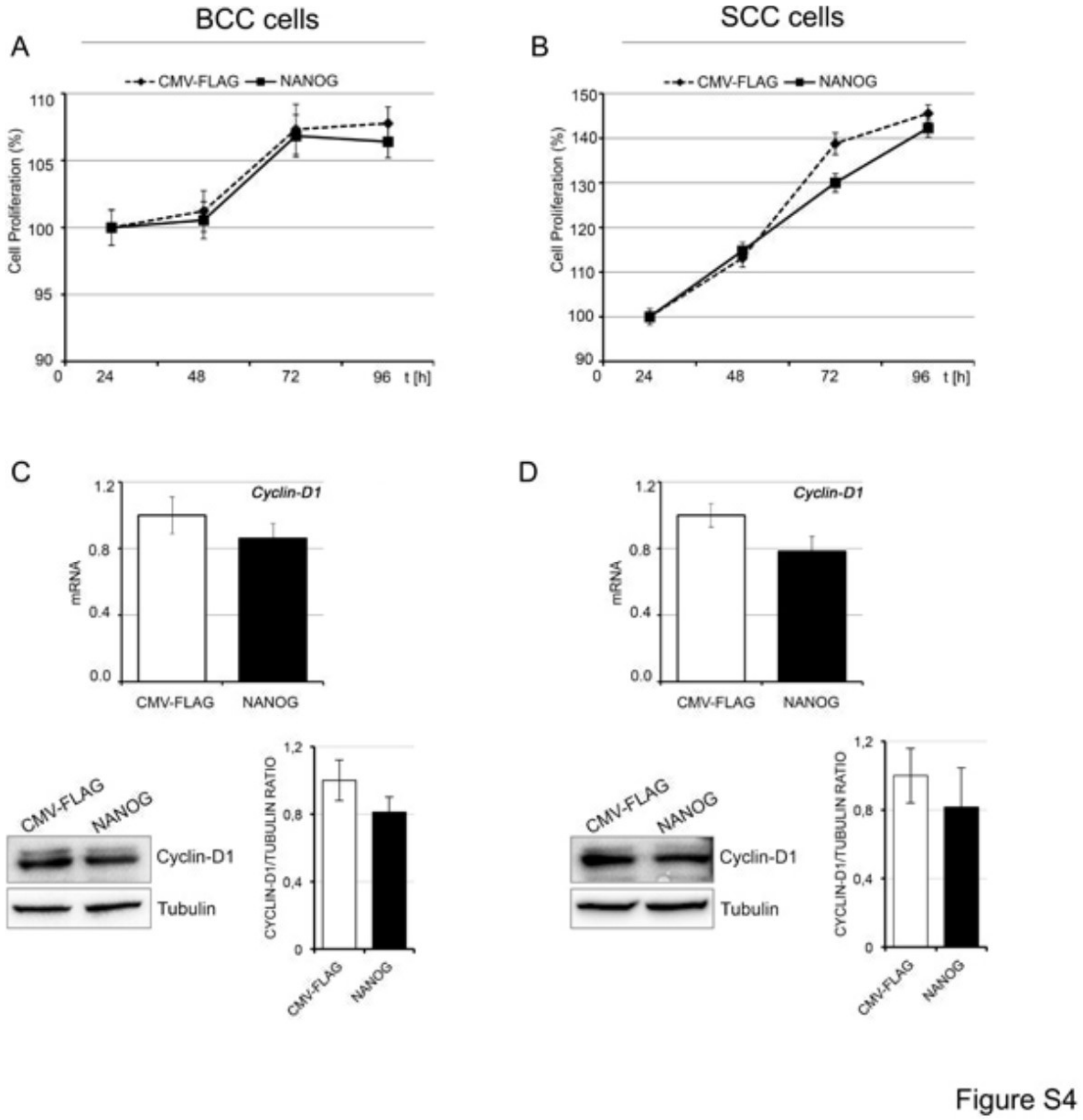

Figure S4. NANOG overexpression does not affect cell proliferation of BCC and SCC cells. (**A, B**) BCC and SCC cells were transfected with NANOG plasmid or the CMV-FLAG, and cell proliferation was assessed by MTT assay after 24, 48, 72 and 96 h. (**C, D**) Cyclin-D1 mRNA and protein levels in BCC cells and SCC cells transfected with NANOG plasmid or CMV-FLAG plasmid. Data represent the mean of 3 independent experiments in duplicate.

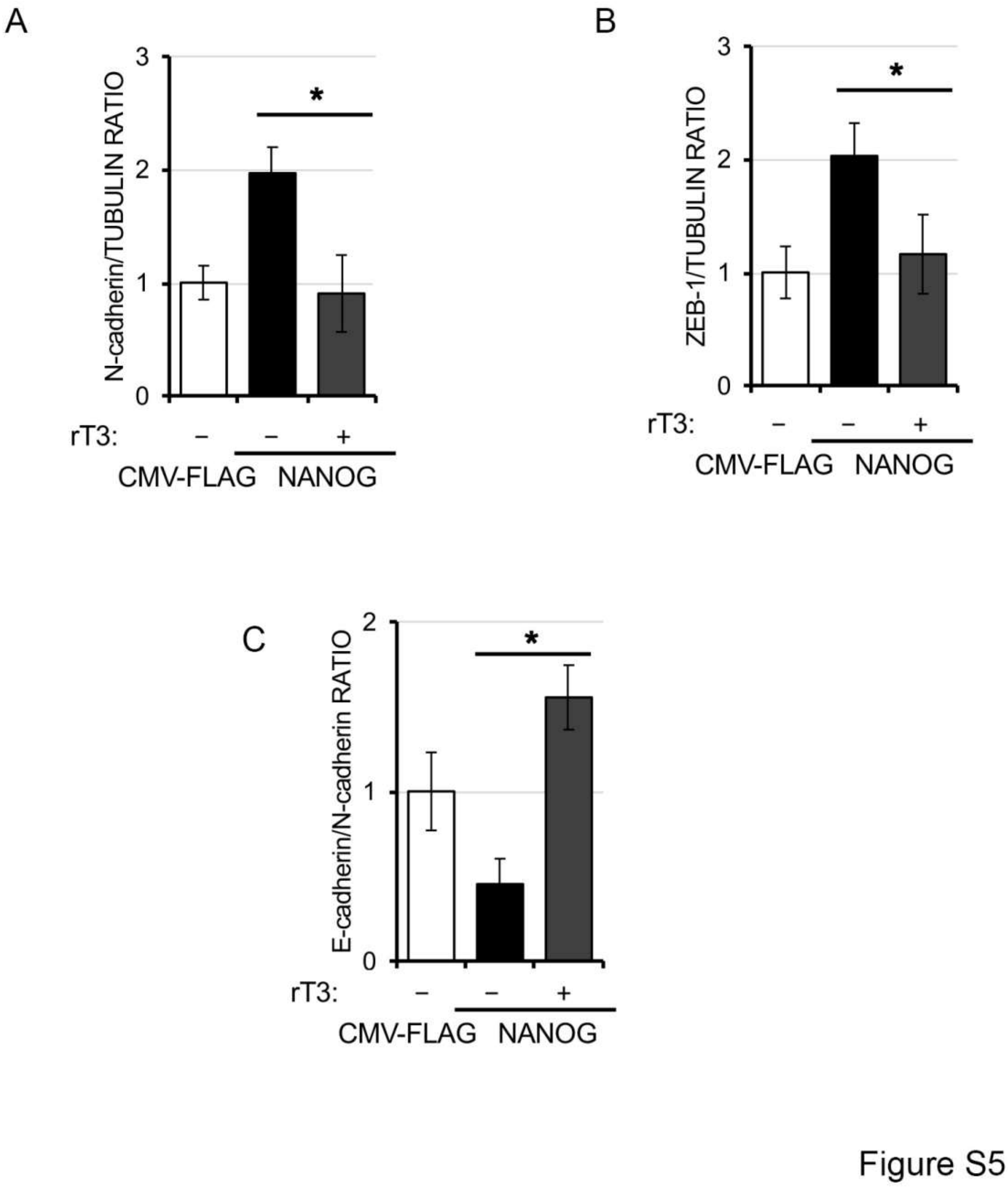

Figure S5. D2 inhibition reduces the migration and EMT of BCC cells induced by NANOG. Quantification of the protein levels of N-cadherin (**A**) and ZEB-1 (**B**) versus Tubulin levels and the E-cadherin/N-cadherin ratio (**C**) in the experiment shown in Figure 5D is represented by histograms. * $p < 0.05$.

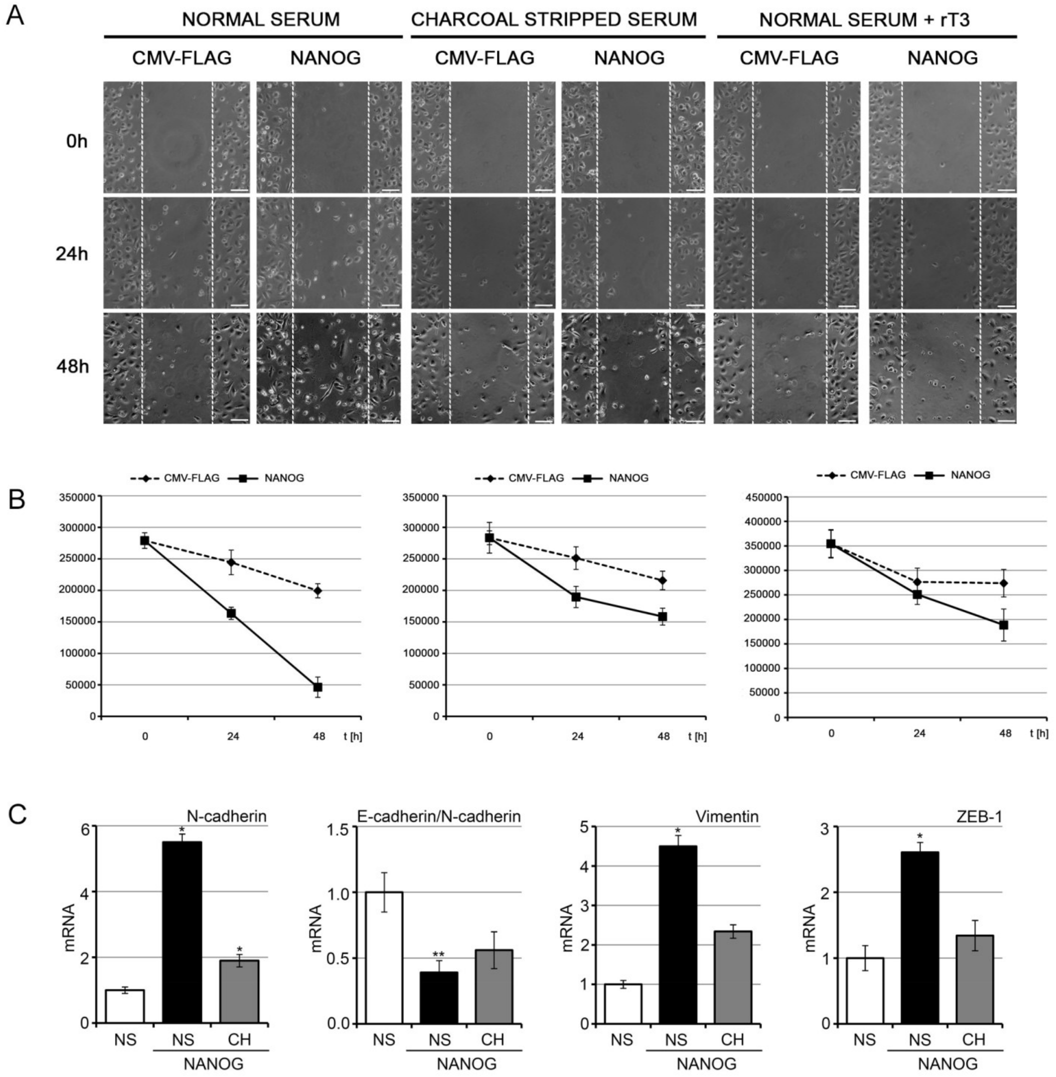

Figure S6

Figure S6. Reduction of TH signaling and D2 activity attenuates EMT of SCC cells. (**A**) Wound scratch assay was performed in SCC13 cells transfected with NANOG plasmid or the CMV-FLAG plasmid. The migration index was measured at 0, 24 and 48 h, under three conditions that include (i) Normal Serum, (ii) Charcoal-Stripped Serum and (iii) Normal Serum + 30.0 nM rT3. Scale bars represent 100 μm. (**B**) Summary graph showing typical wounding area at indicated time points during the scratch wound assay. Data represent the mean of 3 independent experiments in duplicate. (**C**) N-cadherin, E-cadherin/N-cadherin ratio, Vimentin and ZEB-1 mRNA levels in SCC13 cells transfected with NANOG plasmid or the CMV-FLAG plasmid and grown Normal Serum (NS) or Charcoal Stripped Serum (CH). Data represent the mean of 3 independent experiments.

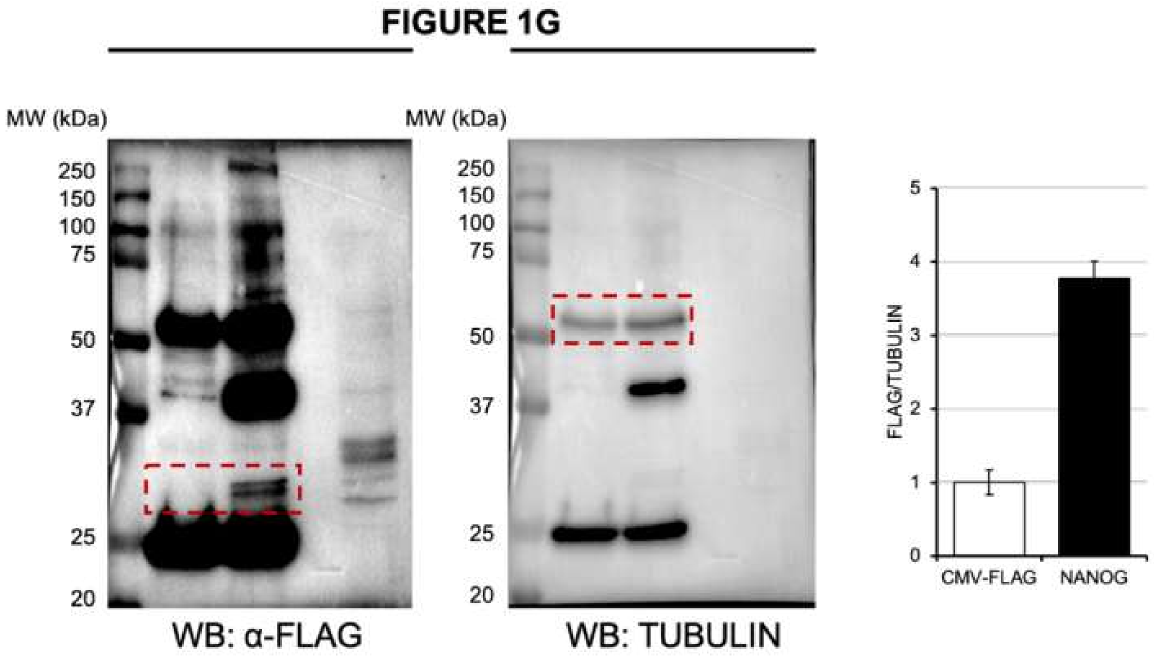

Figure S7. Uncropped images of the blots shown in Figure 1. Molecular weight (MW) in kilodalton (kDa). Quantification of the protein levels of α-FLAG versus TUBULIN levels.

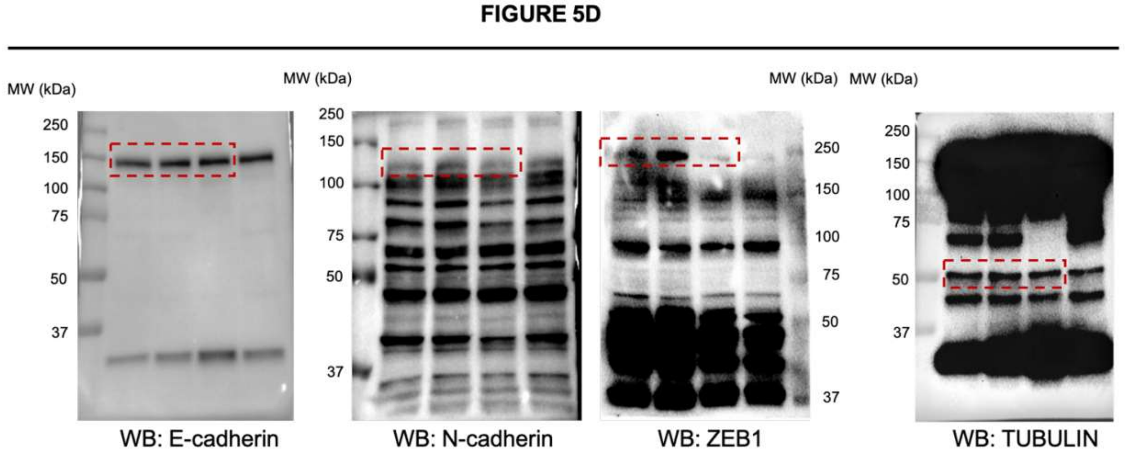

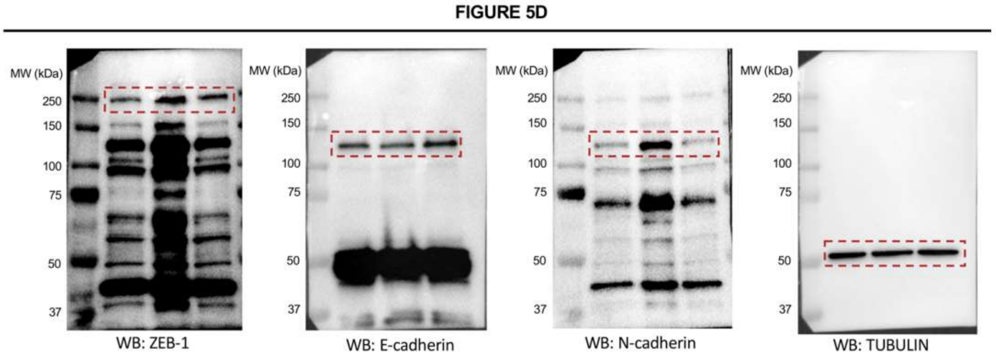

Figure S8. Uncropped images of the blots shown in Figure 5. Molecular weight (MW) in kilodalton (kDa).

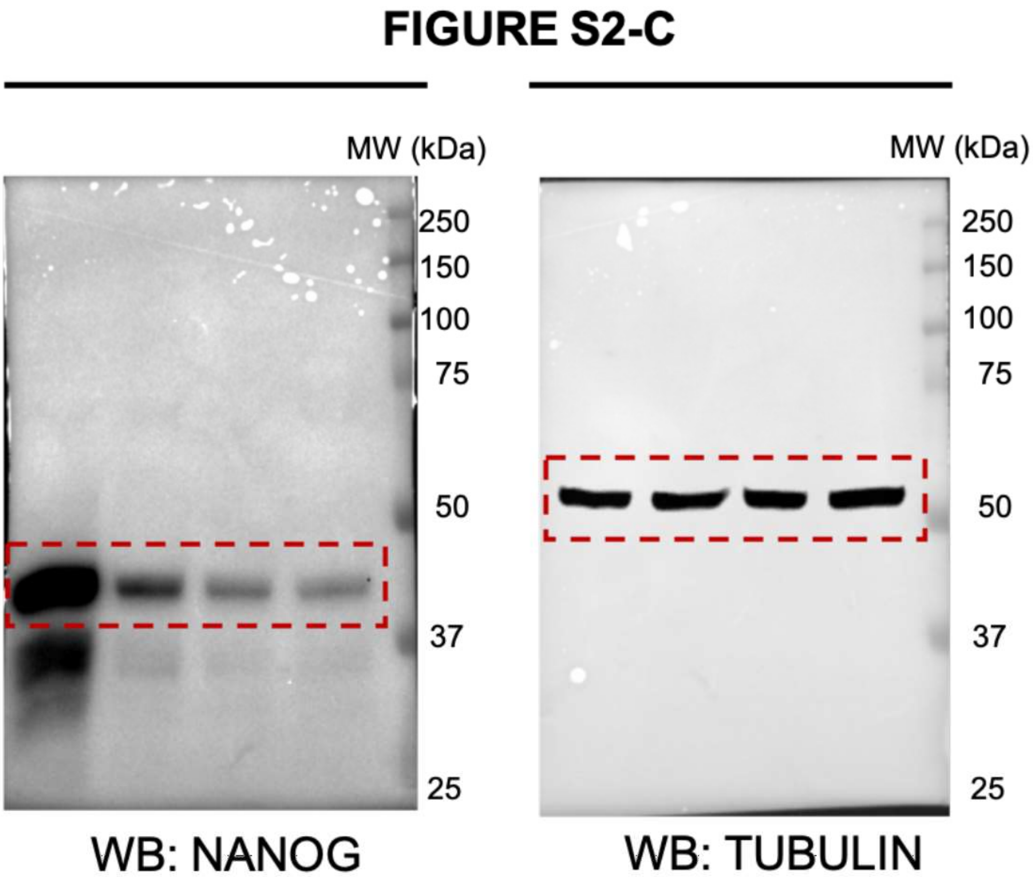

Figure S9. Uncropped images of the blots shown in Figure S2. Molecular weight (MW) in kilodalton (kDa).

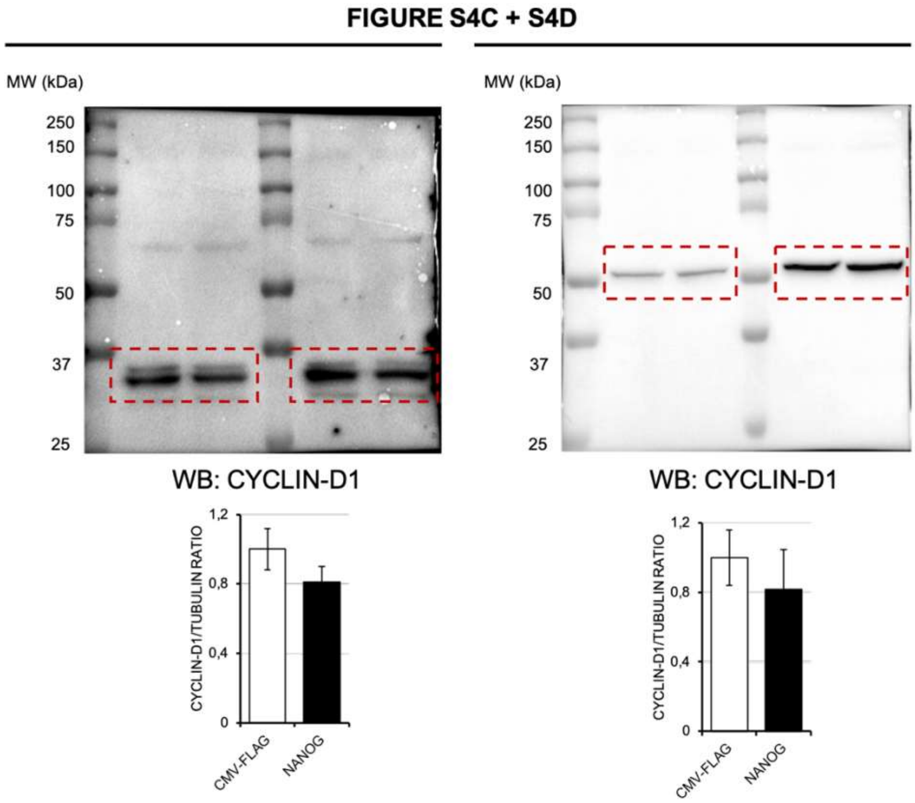

Figure S10. Uncropped images of the blots shown in Figure S3. Molecular weight (MW) in kilodalton (kDa). Quantification of the protein levels of CYCLIN-D1 versus TUBULIN levels.

Table S1. In silico analysis of Transcription Factor Binding Sites (TFBS) ~1.3 kb upstream from the Transcription Start Site (TSS) of Dio2 gene.

Matrix	Detailed Matrix Information	Start Position	End Position	Anchor Position	Strand	Matrix Similarity
V$NACA1.01	Nascent polypeptide-associated complex subunit alpha 1	12	0	6	+	0,95
V$PBX3.01	Pre-B-cell leukemia homeobox 3	28	12	20	+	0,957
V$HIC1.02	Hypermethylated in cancer 1 (secondary DNA binding preference)	35	23	29	+	0,957
V$CSRNP1.01	Cysteine-serine-rich nuclear protein 1 (AXUD1, AXIN1 up-regulated 1)	36	30	33	+	1
V$SPI1.02	SPI-1 proto-oncogene; hematopoietic transcription factor PU.1	50	30	40	+	0,966
V$INSM1.01	Zinc finger protein insulinoma-associated 1 (IA-1) functions as a transcriptional repressor	49	37	43	+	0,949
V$NMP4.01	NMP4 (nuclear matrix protein 4) / CIZ (Cas-interacting zinc finger protein)	61	51	56	+	0,97
V$CEBPE.02	CCAAT/enhancer binding protein (C/EBP), epsilon	66	52	59	-	0,993
V$PEA3.01	Polyomavirus enhancer A binding protein 3, ETV4 (Ets variant gene 4)	77	57	67	-	0,95
V$HMX3.01	H6 homeodomain HMX3/Nkx5.1 transcription factor	92	74	83	-	0,921
V$MZF1.02	Myeloid zinc finger protein MZF1	104	94	99	-	1
V$SPI1.02	SPI-1 proto-oncogene; hematopoietic transcription factor PU.1	110	90	100	-	0,965
V$ZTRE.03	5' half site of ZTRE motif	112	96	104	+	0,984
V$ZBTB7.03	Zinc finger and BTB domain containing 7A, pokemon	118	96	107	+	0,941
V$ZNF219.01	Kruppel-like zinc finger protein 219	120	98	109	+	0,986
V$CKROX.01	Collagen krox protein (zinc finger protein 67 - zfp67)	118	100	109	-	0,909
V$BKLF.02	Kruppel-like factor 3 (basic)	119	101	110	-	0,99
V$MAZ.01	Myc associated zinc finger protein (MAZ)	116	104	110	-	0,951
V$GLIS3.01	GLIS family zinc finger 3, Gli-similar 3	119	103	111	+	0,94

V$ZBED4.02	Zinc finger, BED-type containing 4; polyG binding sites	118	104	111	-	0,951
V$INSM1.01	Zinc finger protein insulinoma-associated 1 (IA-1) functions as a transcriptional repressor	117	105	111	-	0,911
V$ZBP89.01	Zinc finger transcription factor ZBP-89	123	101	112	+	0,959
V$ZTRE.04	3' half site of ZTRE motif	120	104	112	-	0,982
V$PLAG1.02	Pleomorphic adenoma gene 1	130	108	119	-	1
V$WT1.02	Wilms Tumor Suppressor	131	113	122	-	0,951
V$GSH2.01	Homeodomain transcription factor Gsh-2	148	130	139	+	0,966
V$PCE1.01	Photoreceptor conserved element 1	147	131	139	+	0,914
V$MSX.01	Homeodomain proteins MSX-1 and MSX-2	149	131	140	+	0,989
V$EVI1.07	Evi-1 zinc finger protein, carboxy-terminal zinc finger domain	173	157	165	+	0,909
V$STAT5.01	STAT5: signal transducer and activator of transcription 5	178	160	169	-	0,94
V$LEF1.02	TCF/LEF-1, involved in the Wnt signal transduction pathway	177	161	169	+	0,967
V$SMARCA3.01	SWI/SNF related, matrix associated, actin dependent regulator of chromatin, subfamily a, member 3	188	178	183	+	0,967
V$IRF3.01	Interferon regulatory factor 3 (IRF-3)	196	172	184	-	0,949
V$CREL.01	c-Rel	196	182	189	+	0,964
V$CMYB.01	c-Myb, important in hematopoesis, cellular equivalent to avian myoblastosis virus oncogene v-myb	230	210	220	+	0,92
V$NF1.04	Nuclear factor 1	236	216	226	+	0,91
V$HNF6.02	Liver enriched Cut - Homeodomain transcription factor HNF6 (ONECUT1)	241	225	233	-	0,913
V$HBP1.02	HMG box-containing protein 1	249	227	238	+	0,987
**V$NANOG.01**	**Homeobox transcription factor Nanog**	**249**	**231**	**240**	**+**	**0,942**
V$NMP4.01	NMP4 (nuclear matrix protein 4) / CIZ (Cas-interacting zinc finger protein)	266	256	261	+	0,976
V$STAT3.02	Signal transducer and activator of transcription 3	277	259	268	-	0,965
V$HOXB9.01	Abd-B-like homeodomain protein Hoxb-9	282	266	274	+	0,914

V$YY2.01	Transcription factor yin yang 2	286	264	275	+	0,961
V$EVI1.07	Evi-1 zinc finger protein, carboxy-terminal zinc finger domain	285	269	277	-	0,901
V$MOK2.02	Ribonucleoprotein associated zinc finger protein MOK-2 (human)	312	292	302	+	0,992
V$RFX5.01	Regulatory factor X, 5 (influences HLA class II expression)	318	300	309	-	0,981
V$FAC1.01	Fetal Alz-50 clone 1 (FAC1)	321	311	316	-	0,965
V$FOXP1.02	Sites bound by FOXP1 and an alternative splicing variant FOXP1_ES, activated in ESCs	325	309	317	-	1
V$FAC1.01	Fetal Alz-50 clone 1 (FAC1)	324	314	319	-	0,97
V$FOXP1.02	Sites bound by FOXP1 and an alternative splicing variant FOXP1_ES, activated in ESCs	328	312	320	-	1
V$FAC1.01	Fetal Alz-50 clone 1 (FAC1)	327	317	322	-	0,983
V$FOXP1.02	Sites bound by FOXP1 and an alternative splicing variant FOXP1_ES, activated in ESCs	331	315	323	-	1
V$AHRARNT.03	DRE (dioxin response elements), XRE (xenobiotic response elements) bound by AHR/ARNT heterodimers	349	325	337	-	0,962
V$MYRF.01	Myelin regulatory factor	347	335	341	-	0,958
V$GTF3R4.01	GTF2I-like repeat 4 of GTF3	355	345	350	+	0,979
V$CRX.03	Cone-rod homeobox-containing transcription factor	359	343	351	-	0,981
V$PHOX2.01	Phox2a (ARIX) and Phox2b	363	343	353	-	0,949
V$INSM1.01	Zinc finger protein insulinoma-associated 1 (IA-1) functions as a transcriptional repressor	368	356	362	+	0,968
V$TGIF.01	TG-interacting factor belonging to TALE class of homeodomain factors	372	356	364	-	1
V$VMYB.04	v-Myb, AMV v-myb	387	367	377	+	0,993
V$ETV1.02	Ets variant 1	397	377	387	-	0,994
V$IRF4.01	Interferon regulatory factor (IRF)-related protein (NF-EM5, PIP, LSIRF, ICSAT)	413	389	401	+	0,974
V$STAT3.02	Signal transducer and activator of transcription 3	416	398	407	-	0,974

V$STAT.01	Signal transducers and activators of transcription	470	452	461	+	0,914
V$HMX3.02	Hmx3/Nkx5-1 homeodomain transcription factor	471	453	462	-	0,923
V$GKLF.02	Gut-enriched Krueppel-like factor	472	454	463	+	0,976
V$HMX3.02	Hmx3/Nkx5-1 homeodomain transcription factor	476	458	467	+	0,921
V$SMAD.01	Sma- and Mad-related proteins	479	469	474	-	1
V$TH1E47.01	Thing1/E47 heterodimer, TH1 bHLH member specific expression in a variety of embryonic tissues	486	466	476	+	0,94
V$CEBPB.01	CCAAT/enhancer binding protein beta	505	491	498	+	0,958
V$RREB1.01	Ras-responsive element binding protein 1	540	526	533	+	0,94
V$INSM1.01	Zinc finger protein insulinoma-associated 1 (IA-1) functions as a transcriptional repressor	544	532	538	-	0,921
V$VMYB.02	v-Myb	609	589	599	+	0,97
V$FOXP1.01	Forkhead box P1	611	595	603	-	0,985
V$ARE.02	Androgene receptor binding site, IR3 sites	621	603	612	-	0,982
V$MAZ.01	Myc associated zinc finger protein (MAZ)	627	615	621	+	0,91
V$ZNF263.01	Zinc finger protein 263, ZKSCAN12 (zinc finger protein with KRAB and SCAN domains 12)	629	615	622	-	0,949
V$ZNF263.02	Zinc finger protein 263, ZKSCAN12 (zinc finger protein with KRAB and SCAN domains 12)	632	618	625	-	0,97
V$PPARG.02	Peroxisome proliferator-activated receptor gamma	666	644	655	+	0,91
V$IR1_NGRE.01	Repressive binding sites for glucocorticoid receptor (IR1)	675	661	668	+	0,978
V$PLAG1.02	Pleomorphic adenoma gene 1	692	670	681	+	1
V$ZTRE.04	3' half site of ZTRE motif	696	680	688	+	0,984
V$BKLF.02	Kruppel-like factor 3 (basic)	699	681	690	+	0,992
V$ZBED4.02	Zinc finger, BED-type containing 4; polyG binding sites	697	683	690	+	0,922
V$MAZR.01	MYC-associated zinc finger protein related transcription factor	696	684	690	+	0,901

V$CKROX.01	Collagen krox protein (zinc finger protein 67 - zfp67)	700	682	691	+	0,973
V$ZNF263.01	Zinc finger protein 263, ZKSCAN12 (zinc finger protein with KRAB and SCAN domains 12)	698	684	691	-	0,935
V$ZBTB7.03	Zinc finger and BTB domain containing 7A, pokemon	704	682	693	-	0,949
V$ZTRE.03	5' half site of ZTRE motif	704	688	696	-	0,988
V$CREL.01	c-Rel	709	695	702	-	0,957
V$GATA2.02	GATA-binding factor 2	722	710	716	+	0,905
V$ZBTB3.01	Zinc finger and BTB domain containing 3	738	728	733	+	0,997
V$CSRNP1.01	Cysteine-serine-rich nuclear protein 1 (AXUD1, AXIN1 up-regulated 1)	744	738	741	-	1
V$CARF.01	Calcium-reponse factor	752	742	747	+	0,979
O$VTATA.01	Cellular and viral TATA box elements	787	771	779	+	1
V$PIT1.01	Pit1, GHF-1 pituitary specific pou domain transcription factor	789	775	782	-	0,965
V$TEAD4.01	TEA domain family member 4, TEF-3	794	782	788	-	0,942
V$DLX3.01	Distal-less 3 homeodomain transcription factor	805	787	796	-	0,994
V$NOBOX.01	Homeobox containing germ cell-specific transcription factor NOBOX	807	789	798	-	0,991
V$GSH2.01	Homeodomain transcription factor Gsh-2	808	790	799	-	0,975
V$BSX.01	Brain specific homeobox	808	790	799	+	0,974
V$PCE1.01	Photoreceptor conserved element 1	807	791	799	-	0,96
V$NKX12.01	NK1 homeobox 2, Sax1-like	807	791	799	-	0,916
V$S8.01	Binding site for S8 type homeodomains	811	791	801	-	0,995
V$DLX2.01	Distal-less homeobox 2	810	792	801	+	0,929
V$IRF4.01	Interferon regulatory factor (IRF)-related protein (NF-EM5, PIP, LSIRF, ICSAT)	827	803	815	-	0,954
V$CRX.01	Cone-rod homeobox-containing transcription factor / otx-like homeobox gene	835	819	827	-	0,971
V$CEBPB.01	CCAAT/enhancer binding protein beta	840	826	833	+	0,945
V$CREB.02	cAMP-responsive element binding protein	853	833	843	-	0,926
V$E4F.01	GLI-Krueppel-related transcription factor, regulator of adenovirus E4 promoter	850	838	844	+	0,921
V$CREB1.01	cAMP-responsive element binding protein 1	855	835	845	+	1

V$CREB1.01	cAMP-responsive element binding protein 1	856	836	846	-	1
V$CREB.02	cAMP-responsive element binding protein	858	838	848	+	0,927
V$MYT1.02	MyT1 zinc finger transcription factor involved in primary neurogenesis	915	903	909	+	0,9
V$GKLF.02	Gut-enriched Krueppel-like factor	928	910	919	-	0,965
V$ZKSCAN3.01	Zinc finger with KRAB and SCAN domains 3	937	915	926	+	1
V$ZBED4.02	Zinc finger, BED-type containing 4; polyG binding sites	936	922	929	-	0,951
V$ZBP89.01	Zinc finger transcription factor ZBP-89	941	919	930	+	0,935
V$WT1.01	Wilms Tumor Suppressor	941	923	932	-	0,938
V$WT1.02	Wilms Tumor Suppressor	943	925	934	-	0,961
V$SOX6.01	SRY (sex determining region Y)-box 6	960	938	949	+	0,984
V$PLU1_JARID1B.01	Jumonji, AT rich interactive domain 1B	957	949	953	+	0,961
O$MTATA.01	Muscle TATA box	966	950	958	+	0,901
V$GKLF.02	Gut-enriched Krueppel-like factor	971	953	962	-	0,976
V$HOMEZ.01	Homeobox and leucine zipper encoding transcription factor	989	975	982	+	0,963
V$GKLF.02	Gut-enriched Krueppel-like factor	1001	983	992	+	0,966
V$PPARG.03	Peroxisome proliferator-activated receptor gamma, DR1 sites	1005	983	994	+	0,913
V$TGIF.01	TG-interacting factor belonging to TALE class of homeodomain factors	1005	989	997	+	1
V$SMARCA3.02	SWI/SNF related, matrix associated, actin dependent regulator of chromatin, subfamily a, member 3	1036	1026	1031	+	0,986
V$BARX1.01	BARX homeobox 1	1043	1025	1034	-	0,917
V$GSH2.01	Homeodomain transcription factor Gsh-2	1044	1026	1035	-	0,952
V$MSX1.01	Muscle-segment homeobox 1, msh homeobox 1	1044	1026	1035	+	0,907
V$SATB1.01	Special AT-rich sequence-binding protein 1, predominantly expressed in thymocytes, binds to matrix attachment regions (MARs)	1056	1042	1049	-	0,967
V$ZFP652.01	Zinc finger protein 652 (ZNF652)	1063	1049	1056	+	0,909
V$NFAT.01	Nuclear factor of activated T-cells	1068	1050	1059	+	0,965
V$CRX.01	Cone-rod homeobox-containing transcription factor / otx-like homeobox gene	1075	1059	1067	+	0,945

V$GATA2.01	GATA-binding factor 2	1076	1064	1070	+	0,922
V$SALL1.01	Spalt-like transcription factor 1	1082	1070	1076	+	0,961
V$CDX1.01	Intestine specific homeodomain factor CDX-1	1087	1069	1078	-	0,961
V$HOXD10.01	Homeobox D10	1087	1071	1079	+	0,956
V$NKX61.01	NK6 homeobox 1	1087	1073	1080	+	0,915
V$LMX1B.01	LIM-homeodomain transcription factor	1095	1073	1084	+	0,923
V$MTBF.01	Muscle-specific Mt binding site	1095	1087	1091	-	0,902
V$THAP1.01	THAP domain containing, apoptosis associated protein	1100	1090	1095	-	0,924
V$CEBPE.02	CCAAT/enhancer binding protein (C/EBP), epsilon	1103	1089	1096	-	0,974
V$ETV1.02	Ets variant 1	1118	1098	1108	+	0,99
V$STAT3.02	Signal transducer and activator of transcription 3	1121	1103	1112	-	0,959
V$NF1.03	Non-palindromic nuclear factor I binding sites	1127	1107	1117	+	0,995
V$AREB6.01	AREB6 (Atp1a1 regulatory element binding factor 6)	1127	1115	1121	+	0,938
V$IRF1.01	Interferon regulatory factor 1	1141	1117	1129	-	0,952
V$BARX2.01	Barx2, homeobox transcription factor that preferentially binds to paired TAAT motifs	1171	1153	1162	+	0,973
V$HOXC13.01	Homeodomain transcription factor HOXC13	1170	1154	1162	-	0,922
V$HHEX.01	Hematopoietically expressed homeobox, proline-rich homeodomain protein	1175	1157	1166	+	0,969
V$IRF4.01	Interferon regulatory factor (IRF)-related protein (NF-EM5, PIP, LSIRF, ICSAT)	1179	1155	1167	-	0,958
V$NMP4.01	NMP4 (nuclear matrix protein 4) / CIZ (Cas-interacting zinc finger protein)	1178	1168	1173	-	0,972
V$SATB1.01	Special AT-rich sequence-binding protein 1, predominantly expressed in thymocytes, binds to matrix attachment regions (MARs)	1188	1174	1181	+	0,955
V$BARX2.01	Barx2, homeobox transcription factor that preferentially binds to paired TAAT motifs	1193	1175	1184	+	0,953
V$NKX61.02	NK6 homeobox 1	1191	1177	1184	+	0,925
V$KLF7.02	Kruppel-like factor 7 (ubiquitous, UKLF) (secondary DNA binding preference)	1212	1194	1203	-	0,9

V$VMYB.04	v-Myb, AMV v-myb	1215	1195	1205	+	0,907
V$TBX5.01	T-Box factor 5 site (TBX5), mutations related to Holt-Oram syndrome	1220	1192	1206	+	0,99
V$AREB6.02	AREB6 (Atp1a1 regulatory element binding factor 6)	1215	1203	1209	-	0,978
V$CEBPB.02	CCAAT/enhancer binding protein beta	1223	1209	1216	+	0,929
V$NFY.04	Nuclear factor Y (Y-box binding factor)	1229	1215	1222	+	0,923
V$TEAD4.01	TEA domain family member 4, TEF-3	1254	1242	1248	-	0,983
V$SOX1.04	SRY (sex determining region Y)-box 1, dimeric binding sites	1280	1258	1269	-	0,901
V$CPHX.01	Cytoplasmic polyadenylated homeobox	1290	1268	1279	+	0,95
V$GATA.01	GATA binding factor	1291	1279	1285	+	0,998

Table S2. List of oligonucleotides.

Oligo	Name/Gene ID	Sense	Sequence
**Oligonucleotides used for Real-Time PCR**			
Cyclin-D1	*Ccnd1/CCND1*	Forward	GCTCCTGTGCTGCGAAGTGGA
		Reverse	TCATGGCCAGCGGGAAGACCT
Cyclophilin A	*CypA*	Forward	CGCCACTGTCGCTTTTCG
		Reverse	AACTTTGTCTGCAAACAGCTC
CYCLOPHILIN A	*CYPA*	Forward	AGTCCATCTATGGGGAGAAATTTG
		Reverse	GCCTCCACAATATTCATGCCTTC
Dio2	*Dio2*	Forward	CTTCCTCCTAGATGCCTACAAAC
		Reverse	GGCATAATTGTTACCTGATTCAGG
DIO2	*DIO2*	Forward	CTCTATGACTCGGTCATTCTGC
		Reverse	TGTCACCTCCTTCTGTACTGG
E-cadherin	*Cdh1*	Forward	CGTCCTGCCAATCCTGATGA
		Reverse	ACCACTGCCCTCGTAATCGAAC
E-CADHERIN	*CDH1*	Forward	GGCGCCACCTCGAGAGA
		Reverse	TGTCGACCGGTGCAATCTT
LGR5	*Lgr5*	Forward	CAACATCAGTCAGCTACCCG
		Reverse	GTCTCAGCTGGTTGTTCTGC
Nrg1	*Nrg1*	Forward	AACCCACCACCAGAGAATGT
		Reverse	GATGCTTTCTGTGTGCCCAT
N-cadherin	*Cdh2*	Forward	ACAGTGGAGCTCTACAAAGG
		Reverse	CTGAGATGGGGTTGATAATG
N-CADHERIN	*CDH2*	Forward	ACAGTGGCCACCTACAAAGG
		Reverse	CCGAGATGGGGTTGATAATG
Nanog	*Nanog*	Forward	AAGAACTCTCCTCCATTCTGAACCT
		Reverse	GCACTTCATCCTTTGGTTTTGAA
NANOG	*NANOG*	Forward	CTGCTGAGATGCCTCACACG
		Reverse	CTTCCTTTTTTGCGACACTC
NANOG-P8	*NANOGP8*	Forward	CTGCTGAGATGCCTCACACA
		Reverse	CTTCCTTTTTTGCGACACTA
SOX2	*Sox2*	Forward	CTACATGAACGGCTCGCCCACCTAC
		Reverse	CTGGCCTCGGACTTGACCACAGAG
SOX9	*Sox9*	Forward	AGGGCTACGACTGGACGCTGGTG
		Reverse	TGTAATCGGGGTGGTCTTTCTTGTGCT
VIMENTIN	*VIM*	Forward	GAACCTGCAGGAGGCAGAAG
		Reverse	CATCTTAACATTGAGCAGGTC
Vimentin	*Vim*	Forward	GAACCTCCAGGAGGCCGAGG
		Reverse	CATCTTAACATTGAGCAGATC
ZEB1	*Zeb1/ZEB1*	Forward	GCAGAAAATGAGCAAAACCATGA
		Reverse	TGGGTTCTGTATGCAAAGGTG
Oligonucleotides used for ChIP Analysis			
NANOG-ChIP		Forward	GGTAAACTGGATTAGGGACTGGC
		Reverse	GAGGGAGAAAAGCTAAATTAG
Oligonucleotides used for NANOG Binding Site mutation			
pmD2_U	Forward		CGCTCCTGGAGAACCTGGAGAA
NBS_mut_L	Reverse		CACCTTCTTTTTTGCCAGGAAGGTGGTG
NBS_mut_U	Forward		CTCGGAATTGCCGTAATTGATGGGT
pmD2_L	Reverse		GCCGCTCGAGCTTCTCTGCCTCCTCGGTCAGT
Oligonucleotides used for NANOG Silencing			
Sh_NANOG-1	Forward		AATTC GCAAGAACTCTCCTCCATTCT caagaga AGAATGGAGGAGAGTTCTTGC TTTTTC

	Reverse	TCGA gaaaaa GCAAGAACTCTCCTCCATTCT tctcttg AGAATGGAGGAGAGTTCTTGC g
Sh_NANOG-2	Forward	AATTC GGACCAACCCAACTTGGAACA caagaga TGTTCCAAGTTGGGTTGGTCC TTTTTC
	Reverse	TCGA gaaaaa GGACCAACCCAACTTGGAACA tctcttg TGTTCCAAGTTGGGTTGGTCC g

© 2020 by the authors. Licensee MDPI, Basel, Switzerland. This article is an open access article distributed under the terms and conditions of the Creative Commons Attribution (CC BY) license (http://creativecommons.org/licenses/by/4.0/).
